# 3,3′-(Butane-1,4-di­yl)diimidazole-1,1′-diium bis­(triiodide)

**DOI:** 10.1107/S1600536808022393

**Published:** 2008-07-19

**Authors:** Ai-E. Shi, Ying-Hui Yu, Guang-Feng Hou, Jin-Sheng Gao

**Affiliations:** a College of Chemistry and Materials Science, Heilongjiang University, Harbin 150080, People’s Republic of China

## Abstract

The cations and anions of the salt, C_10_H_16_N_4_^2+^·2I_3_^−^, are linked by N—H⋯I hydrogen bonds and π–π stacking inter­actions(with interplanar distances of 3.575 and 3.528 Å) into a three-dimensional supra­molecular network. The asymmetric unit contains two anions and two half-cations; each cation is centrosymmetric.

## Related literature

For literature on 1,1′-(1,4-butanedi­yl)diimidazole, see: Ma *et al.* (2003 ▶). For the structure of another 1,1′-(1,4-butanedi­yl)diimidazole-3,3′-diium salt, see: Yu *et al.* (2008 ▶).
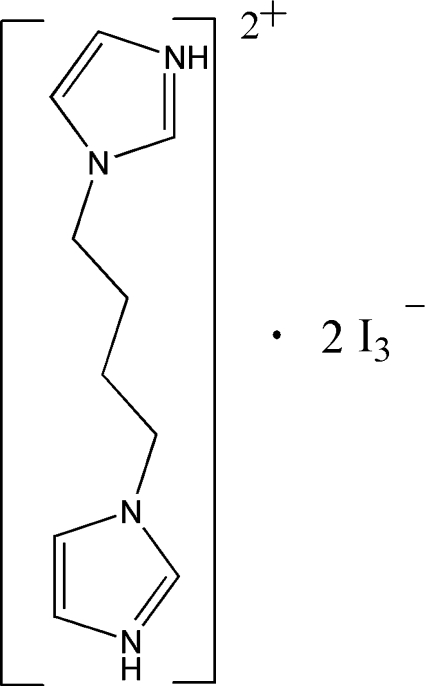


## Experimental

### 

#### Crystal data

C_10_H_16_N_4_^2+^·2I_3_^−^
*M* *_r_* = 953.67
Triclinic, 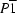

*a* = 8.4753 (17) Å
*b* = 9.7177 (19) Å
*c* = 14.110 (3) Å
α = 95.77 (3)°
β = 92.82 (3)°
γ = 107.17 (3)°
*V* = 1100.9 (4) Å^3^
*Z* = 2
Mo *K*α radiation
μ = 8.46 mm^−1^
*T* = 291 (2) K
0.21 × 0.20 × 0.18 mm

#### Data collection

Rigaku R-AXIS RAPID diffractometer
Absorption correction: multi-scan (*ABSCOR*; Higashi, 1995 ▶) *T*_min_ = 0.266, *T*_max_ = 0.306 (expected range = 0.189–0.218)
8492 measured reflections
3831 independent reflections
3045 reflections with *I* > 2σ(*I*)
*R*_int_ = 0.030

#### Refinement

*R*[*F* ^2^ > 2σ(*F* ^2^)] = 0.035
*wR*(*F* ^2^) = 0.087
*S* = 1.10
3831 reflections
178 parameters
H-atom parameters constrained
Δρ_max_ = 1.19 e Å^−3^
Δρ_min_ = −0.86 e Å^−3^

### 

Data collection: *RAPID-AUTO* (Rigaku, 1998 ▶); cell refinement: *RAPID-AUTO*; data reduction: *CrystalStructure* (Rigaku/MSC, 2002 ▶); program(s) used to solve structure: *SHELXS97* (Sheldrick, 2008 ▶); program(s) used to refine structure: *SHELXL97* (Sheldrick, 2008 ▶); molecular graphics: *SHELXTL* (Sheldrick, 2008 ▶); software used to prepare material for publication: *SHELXL97*.

## Supplementary Material

Crystal structure: contains datablocks global, I. DOI: 10.1107/S1600536808022393/ng2463sup1.cif

Structure factors: contains datablocks I. DOI: 10.1107/S1600536808022393/ng2463Isup2.hkl

Additional supplementary materials:  crystallographic information; 3D view; checkCIF report

## Figures and Tables

**Table 1:** Hydrogen-bond geometry (Å, °)

*D*—H⋯*A*	*D*—H	H⋯*A*	*D*⋯*A*	*D*—H⋯*A*
N2—H11⋯I3^i^	0.85	3.14	3.690 (9)	124
N2—H11⋯I1^ii^	0.85	2.99	3.666 (9)	138
N4—H3⋯I1^iii^	0.86	3.25	3.714 (9)	116
N4—H3⋯I6^iv^	0.86	3.03	3.679 (8)	134

Symmetry codes: (i) 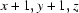; (ii) 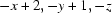; (iii) 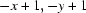, 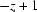; (iv) 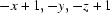.

**Fig. 1.**
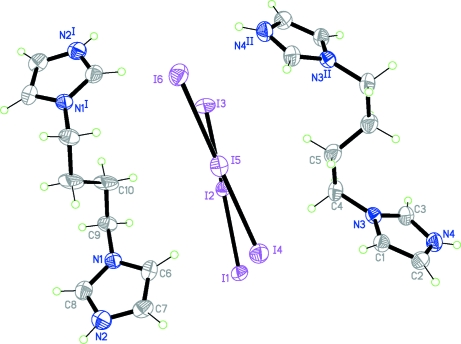
The molecular structure of (I), showing displacement ellipsoids at the 30% probability level for non-H atoms.

**Fig. 2.**
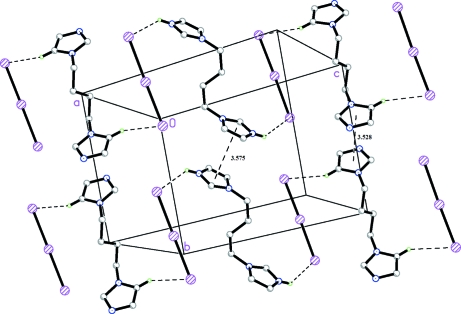
A partial packing view, showing π-π interactions. Dashed lines indicate the hydrogen-bonding interactions and π-π interactions. H atoms not involved in hydrogen bonds have been omitted for clarity.

## supplementary crystallographic information

### Comment

The 1,1'-(1,4-butanediyl)diimidazole can be used as a flexible ligand to construct coordination polymer materials (Ma *et al.*, 2003; Yu *et al.*, 2008). In our attempt to synthesize the copper(I) iodide complex with the 1,1'-(1,4-butanediyl)diimidazole, we unexpectedly obtained the title compound (I). Herein, we report its crystal structure.

The asymmetric unit of (I) consists of two halfs of two independent centrosymmetric molecules of 1,1'-(1,4-Butanediyl)diimidazole-3,3'-diium cation and two triiodide anions (Figure 1). The remarkable π-π stacking interactions are observed, with the distance between the π-π stacking planes are 3.575 Å (cations); 3.528 Å (Figure 2),

In the crystal, intermolecular N—H···I, C—H···I hydrogen bonds and π-π stacking interactions link all cations and anions into three-dimensional supramolecular network (Table 1).

### Experimental

1,1'-(1,4-Butanediyl)diimidazole was prepared of imidazole and 1,4-dibromobutane in dimethylsulfoxide solution (Ma *et al.*., 2003). CuI (0.380 g, 2 mmol) and 1,1'-(1,4-butanediyl)diimidazole (0.380 g, 2 mmol) were dissolved in hot methanol solution (15 ml) and added two drops hydrochloric acid then a clear solution was obtained. The resulting solution was allowed to stand in a desiccator at room temperature for several days. Yellow crystals of (I) were obtained. Unexpectedly, the salt-type adducts of this ligands was crystallized from solution.

### Refinement

H atoms bound to C atoms were placed in calculated positions and treated as riding on their parent atoms, with C—H = 0.93 Å (Caromatic); C—H = 0.97 Å (methylene) and with *U*_iso_(H) = 1.2*U*_eq_(C). The N-bound H atoms were located in a difference Fourier map and refined with N—H = 0.85 Å, *U*_iso_(H) = 1.2*U*_eq_(N).  The final different Fourier map had a large peak in the vicinity of the iodine atoms.

### Crystal data

**Table:**

C_10_H_16_N_4_^2+^·2I_3_^–^	*Z* = 2
*M**_r_* = 953.67	*F*_000_ = 844
Triclinic, *P*1	*D*_x_ = 2.877 Mg m^−^^3^
Hall symbol: -P 1	Mo *K*α radiation λ = 0.71073 Å
*a* = 8.4753 (17) Å	Cell parameters from 7434 reflections
*b* = 9.7177 (19) Å	θ = 3.0–27.5º
*c* = 14.110 (3) Å	µ = 8.46 mm^−^^1^
α = 95.77 (3)º	*T* = 291 (2) K
β = 92.82 (3)º	Block, yellow
γ = 107.17 (3)º	0.21 × 0.20 × 0.18 mm
*V* = 1100.9 (4) Å^3^	

### Data collection

**Table:**

Rigaku R-AXIS RAPID diffractometer	3831 independent reflections
Radiation source: fine-focus sealed tube	3045 reflections with *I* > 2σ(*I*)
Monochromator: graphite	*R*_int_ = 0.031
*T* = 291(2) K	θ_max_ = 25.0º
ω scans	θ_min_ = 3.0º
Absorption correction: multi-scan(ABSCOR; Higashi, 1995)	*h* = −10→10
*T*_min_ = 0.266, *T*_max_ = 0.307	*k* = −11→11
8492 measured reflections	*l* = −16→16

### Refinement

**Table:**

Refinement on *F*^2^	Secondary atom site location: difference Fourier map
Least-squares matrix: full	Hydrogen site location: inferred from neighbouring sites
*R*[*F*^2^ > 2σ(*F*^2^)] = 0.035	H-atom parameters constrained
*wR*(*F*^2^) = 0.087	*w* = 1/[σ^2^(*F*_o_^2^) + (0.0254*P*)^2^ + 5.2161*P*] where *P* = (*F*_o_^2^ + 2*F*_c_^2^)/3
*S* = 1.10	(Δ/σ)_max_ = 0.001
3831 reflections	Δρ_max_ = 1.19 e Å^−^^3^
178 parameters	Δρ_min_ = −0.86 e Å^−^^3^
Primary atom site location: structure-invariant direct methods	Extinction correction: none

### Special details

**Table:**

Geometry. All e.s.d.'s (except the e.s.d. in the dihedral angle between two l.s. planes) are estimated using the full covariance matrix. The cell e.s.d.'s are taken into account individually in the estimation of e.s.d.'s in distances, angles and torsion angles; correlations between e.s.d.'s in cell parameters are only used when they are defined by crystal symmetry. An approximate (isotropic) treatment of cell e.s.d.'s is used for estimating e.s.d.'s involving l.s. planes.
Refinement. Refinement of *F*^2^ against ALL reflections. The weighted *R*-factor *wR* and goodness of fit *S* are based on *F*^2^, conventional *R*-factors *R* are based on *F*, with *F* set to zero for negative *F*^2^. The threshold expression of *F*^2^ > σ(*F*^2^) is used only for calculating *R*-factors(gt) *etc*. and is not relevant to the choice of reflections for refinement. *R*-factors based on *F*^2^ are statistically about twice as large as those based on *F*, and *R*- factors based on ALL data will be even larger.

### Fractional atomic coordinates and isotropic or equivalent isotropic displacement parameters (Å^2^)

**Table:**

	*x*	*y*	*z*	*U*_iso_*/*U*_eq_	
C1	0.6046 (11)	0.3684 (10)	0.5506 (6)	0.049 (2)	
H1	0.7142	0.3830	0.5369	0.058*	
C2	0.5511 (12)	0.4469 (11)	0.6176 (7)	0.055 (2)	
H2	0.6159	0.5268	0.6585	0.066*	
C3	0.3377 (12)	0.2777 (12)	0.5469 (7)	0.056 (2)	
H4	0.2293	0.2191	0.5305	0.067*	
C4	0.4772 (13)	0.1552 (11)	0.4282 (6)	0.057 (3)	
H6	0.5458	0.2028	0.3808	0.069*	
H5	0.3664	0.1090	0.3977	0.069*	
C5	0.5466 (12)	0.0400 (11)	0.4628 (7)	0.062 (3)	
H8	0.5474	−0.0293	0.4085	0.075*	
H7	0.6605	0.0860	0.4884	0.075*	
C6	1.0068 (12)	0.3316 (11)	0.1048 (7)	0.055 (2)	
H9	0.9362	0.2915	0.1499	0.066*	
C7	1.1312 (11)	0.4573 (11)	0.1195 (8)	0.057 (3)	
H10	1.1636	0.5208	0.1758	0.068*	
C8	1.1230 (13)	0.3611 (11)	−0.0302 (7)	0.056 (3)	
H12	1.1490	0.3477	−0.0930	0.067*	
C9	0.8858 (8)	0.1352 (8)	−0.0341 (6)	0.055 (2)	
H13	0.8975	0.1278	−0.1023	0.066*	
H14	0.7734	0.1358	−0.0247	0.066*	
C10	0.9139 (8)	0.0029 (8)	0.0049 (6)	0.079 (4)	
H15	0.8923	0.0055	0.0718	0.094*	
H16	0.8364	−0.0844	−0.0293	0.094*	
I1	0.55146 (9)	0.37444 (7)	0.15451 (5)	0.05732 (19)	
I2	0.54230 (7)	0.06704 (6)	0.16015 (4)	0.04222 (15)	
I3	0.53942 (8)	−0.22804 (7)	0.16387 (5)	0.0602 (2)	
I4	0.96410 (8)	0.34498 (8)	0.37380 (5)	0.0608 (2)	
I5	0.99004 (7)	0.05335 (7)	0.33765 (4)	0.05118 (17)	
I6	1.03499 (9)	−0.23842 (8)	0.31071 (5)	0.0636 (2)	
N1	1.0024 (9)	0.2733 (8)	0.0121 (5)	0.0471 (18)	
N2	1.1981 (10)	0.4712 (9)	0.0356 (6)	0.060 (2)	
H11	1.2906	0.5258	0.0211	0.072*	
N3	0.4708 (8)	0.2639 (8)	0.5063 (5)	0.0443 (17)	
N4	0.3853 (10)	0.3886 (9)	0.6146 (6)	0.058 (2)	
H3	0.3111	0.4094	0.6480	0.070*	

### Atomic displacement parameters (Å^2^)

**Table:**

	*U*^11^	*U*^22^	*U*^33^	*U*^12^	*U*^13^	*U*^23^
C1	0.039 (5)	0.053 (6)	0.048 (5)	0.005 (4)	0.007 (4)	0.002 (5)
C2	0.054 (6)	0.052 (6)	0.049 (6)	0.006 (5)	−0.003 (5)	−0.002 (5)
C3	0.050 (6)	0.068 (7)	0.052 (6)	0.021 (5)	0.009 (5)	0.007 (5)
C4	0.062 (6)	0.074 (7)	0.038 (5)	0.027 (6)	0.005 (5)	−0.001 (5)
C5	0.060 (6)	0.063 (7)	0.067 (7)	0.023 (6)	0.017 (5)	0.002 (5)
C6	0.052 (6)	0.067 (7)	0.049 (6)	0.026 (6)	0.004 (5)	−0.003 (5)
C7	0.039 (5)	0.049 (6)	0.072 (7)	0.005 (5)	−0.010 (5)	−0.012 (5)
C8	0.067 (6)	0.061 (7)	0.050 (6)	0.034 (6)	0.006 (5)	0.011 (5)
C9	0.051 (5)	0.045 (6)	0.060 (6)	0.003 (5)	−0.006 (5)	0.006 (5)
C10	0.056 (6)	0.037 (6)	0.121 (10)	−0.018 (5)	−0.001 (7)	0.009 (6)
I1	0.0714 (4)	0.0472 (4)	0.0598 (4)	0.0265 (3)	0.0108 (3)	0.0079 (3)
I2	0.0397 (3)	0.0447 (3)	0.0383 (3)	0.0090 (3)	0.0015 (2)	−0.0012 (2)
I3	0.0601 (4)	0.0393 (4)	0.0767 (5)	0.0106 (3)	−0.0034 (3)	0.0040 (3)
I4	0.0584 (4)	0.0562 (4)	0.0616 (4)	0.0098 (3)	0.0076 (3)	−0.0007 (3)
I5	0.0415 (3)	0.0630 (4)	0.0443 (3)	0.0107 (3)	−0.0010 (3)	0.0020 (3)
I6	0.0660 (4)	0.0729 (5)	0.0604 (4)	0.0338 (4)	0.0089 (3)	0.0062 (4)
N1	0.051 (4)	0.048 (5)	0.049 (5)	0.022 (4)	0.004 (4)	0.014 (4)
N2	0.049 (5)	0.061 (6)	0.075 (6)	0.024 (4)	0.006 (5)	0.017 (5)
N3	0.040 (4)	0.048 (5)	0.043 (4)	0.009 (4)	0.003 (3)	0.008 (3)
N4	0.054 (5)	0.065 (6)	0.055 (5)	0.015 (4)	0.015 (4)	0.004 (4)

### Geometric parameters (Å, °)

**Table:**

C1—C2	1.335 (13)	C7—N2	1.340 (13)
C1—N3	1.354 (11)	C7—H10	0.9300
C1—H1	0.9300	C8—N1	1.332 (12)
C2—N4	1.348 (12)	C8—N2	1.324 (12)
C2—H2	0.9300	C8—H12	0.9300
C3—N4	1.317 (12)	C9—N1	1.477 (10)
C3—N3	1.326 (11)	C9—C10	1.5246
C3—H4	0.9300	C9—H13	0.9700
C4—N3	1.462 (11)	C9—H14	0.9700
C4—C5	1.519 (13)	C10—C10^ii^	1.489
C4—H6	0.9700	C10—H15	0.9700
C4—H5	0.9700	C10—H16	0.9700
C5—C5^i^	1.487 (19)	I1—I2	2.9745 (10)
C5—H8	0.9700	I2—I3	2.8662 (10)
C5—H7	0.9700	I4—I5	2.9031 (11)
C6—C7	1.347 (13)	I5—I6	2.9604 (11)
C6—N1	1.367 (11)	N2—H11	0.8544
C6—H9	0.9300	N4—H3	0.8628
			
C2—C1—N3	107.7 (8)	N1—C8—H12	127.1
C2—C1—H1	126.2	N2—C8—H12	127.1
N3—C1—H1	126.2	N1—C9—C10	112.9
C1—C2—N4	107.1 (9)	N1—C9—H13	109.0
C1—C2—H2	126.5	C10—C9—H13	109.0
N4—C2—H2	126.5	N1—C9—H14	109.0
N4—C3—N3	108.2 (9)	C10—C9—H14	109.0
N4—C3—H4	125.9	H13—C9—H14	107.8
N3—C3—H4	125.9	C10^ii^—C10—C9	112.0 (5)
N3—C4—C5	112.1 (8)	C10^ii^—C10—H15	109.2
N3—C4—H6	109.2	C9—C10—H15	109.2
C5—C4—H6	109.2	C10^ii^—C10—H16	109.2
N3—C4—H5	109.2	C9—C10—H16	109.2
C5—C4—H5	109.2	H15—C10—H16	107.9
H6—C4—H5	107.9	I3—I2—I1	178.89 (3)
C5^i^—C5—C4	114.5 (10)	I4—I5—I6	176.21 (3)
C5^i^—C5—H8	108.6	C8—N1—C6	108.7 (9)
C4—C5—H8	108.6	C8—N1—C9	125.1 (8)
C5^i^—C5—H7	108.6	C6—N1—C9	126.2 (8)
C4—C5—H7	108.6	C8—N2—C7	112.3 (9)
H8—C5—H7	107.6	C8—N2—H11	114.7
C7—C6—N1	108.1 (9)	C7—N2—H11	131.3
C7—C6—H9	125.9	C3—N3—C1	108.0 (8)
N1—C6—H9	125.9	C3—N3—C4	127.3 (8)
C6—C7—N2	105.0 (9)	C1—N3—C4	124.6 (7)
C6—C7—H10	127.5	C3—N4—C2	109.0 (8)
N2—C7—H10	127.5	C3—N4—H3	118.4
N1—C8—N2	105.9 (9)	C2—N4—H3	132.6

Symmetry codes: (i) −*x*+1, −*y*, −*z*+1; (ii) −*x*+2, −*y*, −*z*.

### Hydrogen-bond geometry (Å, °)

**Table:**

*D*—H···*A*	*D*—H	H···*A*	*D*···*A*	*D*—H···*A*
N2—H11···I3^iii^	0.85	3.14	3.690 (9)	124
N2—H11···I1^iv^	0.85	2.99	3.666 (9)	138
N4—H3···I1^v^	0.86	3.25	3.714 (9)	116
N4—H3···I6^i^	0.86	3.03	3.679 (8)	134
C6—H9···I4	0.93	3.13	3.823 (10)	132

Symmetry codes: (iii) *x*+1, *y*+1, *z*; (iv) −*x*+2, −*y*+1, −*z*; (v) −*x*+1, −*y*+1, −*z*+1; (i) −*x*+1, −*y*, −*z*+1.
